# Descriptive Sensory Analysis of Gluten-Containing and Gluten-Free Chocolate Chip Cookies Available in the Marketplace

**DOI:** 10.3390/foods14132233

**Published:** 2025-06-25

**Authors:** Eniola Ola, Victoria J. Hogan, Han-Seok Seo

**Affiliations:** Department of Food Science, University of Arkansas, 2650 N. Young Avenue, Fayetteville, AR 72704, USA

**Keywords:** gluten, gluten-free, cookie, sensory, descriptive analysis, lexicon, crispy, chewy

## Abstract

Limited research has systematically compared the detailed sensory profiles of commercially available gluten-containing (C) and gluten-free (F) cookies using trained panelists. This study aimed to develop a comprehensive sensory lexicon for C and F chocolate chip cookies and identify key sensory attributes that differentiate them. Seven professionally trained panelists created a lexicon of 33 attributes spanning aroma, flavor, basic taste, texture, and residual property. Using this lexicon, a descriptive analysis was conducted on 12 C and 12 F cookie samples. Multivariate analysis of variance revealed significant differences between the two groups across the 33 sensory attributes (*p* < 0.05). A mixed model analysis showed that C cookies had higher intensities of chocolate-related and sweet aroma complex notes, while F cookies exhibited stronger nutty, artificial, and off-note flavors. In terms of texture, F cookies were higher in toothpack and powdery mouthcoat, while C cookies displayed more melt-in-mouth characteristics. Principal component analysis and agglomerative hierarchical clustering revealed three distinct clusters of test samples within both crispy and chewy cookie types, with some F cookies closely aligning with C profiles. These findings, along with the developed lexicon, provide a valuable foundation for enhancing the sensory appeal and quality of gluten-free chocolate chip cookies.

## 1. Introduction

Gluten is a plant-based protein primarily composed of two fractions, gliadin and glutenin, found in cereal grains such as wheat, barley, rye, and oats [1]. In wheat, gluten functions as a natural binding agent, imparting viscoelasticity, enhancing water absorption, and increasing dough strength [2]. These are key properties that contribute significantly to the overall quality of baked goods [3]. Its heat stability and cohesive characteristics make gluten a valuable functional ingredient in the food industry, where it is widely employed as a binding and extending agent to improve texture and moisture retention in processed foods [3].

Despite its functional advantages, gluten poses significant health risks for individuals with gluten-related disorders. Celiac disease, the most common of these conditions, affects approximately 1% of the global population, with prevalence rates ranging from 0.5% to 2% [4]. As the condition is triggered by gluten ingestion, strict adherence to a gluten-free diet remains the only effective treatment [4]. As a result, gluten-free grains such as rice, maize, quinoa, buckwheat, sorghum, and millet have become increasingly important in the development of gluten-free products [5]. Regulatory standards, such as the U.S. Food and Drug Administration (FDA)’s definition of gluten-free products as containing less than 20 parts per million (ppm) of gluten, ensure product safety and consistency for individuals with gluten-related disorders [6].

The gluten-free market has emerged as one of the fastest-growing segments within the health food sector [7]. Over the past decade, market growth has been driven by improved diagnostic tools for celiac disease, increased awareness of gluten-related conditions, and broader trends toward health-conscious lifestyles [8]. Once limited to specialty health food stores, gluten-free products are now widely available, with nearly 1000 new food and beverage items launched in the U.S. in recent years [9]. By 2032, the global gluten-free market is expected to exceed 14 billion USD, more than doubling its 2022 valuation [9]. This growth is fueled not only by medical necessity but also by non-celiac consumers who perceive gluten-free diets as beneficial for weight loss and general health [10,11]. Emotional, social, and lifestyle factors also contribute to the popularity of gluten-free diets [12,13]. As a result, there is a growing demand for convenient and diverse gluten-free options across product categories such as bread, pasta, snacks, and fast foods [8,12].

However, the development of gluten-free baked goods presents considerable challenges, particularly in achieving acceptable sensory quality. The absence of gluten often results in poor dough consistency, limited gas retention, lower loaf volume, crumbly texture, pale color, dry mouthfeel, and shortened shelf life [14,15,16,17,18,19,20]. While alternative flours such as buckwheat, legumes, maize, and rice are commonly used, they often fall short in replicating the flavor and texture of wheat-based products, resulting in less desirable sensory characteristics [5,21,22]. Flavor and texture, in particular, strongly influence consumer purchasing behavior, especially for staple products such as bread and dairy items [23,24,25,26]. Therefore, enhancing sensory quality is essential to improving consumer acceptance. Addressing these limitations requires integrating sensory evaluation throughout the product development process from ingredient selection to process optimization [27]. Poor sensory characteristics and limited product availability remain barriers to dietary adherence among individuals with celiac disease [28].

Although sensory quality is a critical determinant of consumer satisfaction and purchase behavior, it has historically been underemphasized in gluten-free product development [27]. While research in this domain has grown from just 17 publications in 2012 to approximately 105 in 2022 [29], the focus has largely been on technological, compositional, or nutritional aspects rather than sensory properties [1,5,17,25,30,31], as noted by Capriles et al. [29]. In the past decade, gluten-free baked goods have increasingly been evaluated through discriminative, descriptive, and affective sensory methods. Affective tests, such as consumer acceptance testing using structured 5-, 7-, and 9-point scales, have been commonly employed to assess consumer liking of gluten-free samples such as bread, cakes/brownies/muffins, and cookies/biscuits/crackers [29]. However, most of these studies have been conducted in controlled laboratory settings. To better reflect real-world consumer experiences, future research should incorporate central location and home-use testing.

In contrast, relatively few studies have used descriptive sensory analysis methods, such as quantitative descriptive analysis, flash profiling, and free-choice profiling. These methods typically involve trained panelists to generate detailed sensory profiles [29,32,33]. Among gluten-free baked goods, bread has received the most attention in descriptive sensory studies [34,35,36]. Studies focusing on gluten-free cookies are comparatively scarce and usually involve experimental rather than commercially available formulations [4,32,37,38,39]. This disconnect between laboratory-based research and market products highlights the need for industry-relevant sensory research that reflects the actual consumer experience [4,40].

A foundational component of descriptive sensory analysis is the creation of a standardized sensory lexicon, a set of clearly defined terms used to describe specific sensory attributes [41]. Sensory lexicons serve multiple roles in product development, quality assurance, shelf life monitoring, and process optimization [27,41,42,43]. Sensory lexicons have been developed for various gluten-free products, including bread [44], pasta [45], brownies [46], biscuits [47], crackers [48], rice cakes [49], and cookies [37,38,39]. For example, Da Silva and Conti-Silva [38] developed a lexicon for five chocolate cookies featuring twelve descriptors: two for appearance, two for aroma, three for flavor, and five for texture. However, a comprehensive lexicon specifically for gluten-free cookies, biscuits, and crackers is still lacking. Although Jambrec et al. [33] compiled descriptors for six commercial cookies and one pilot-produced gluten-free sample using a rapid method (free-choice profiling), further research is needed to develop a robust, standardized vocabulary based on a broader and more representative product selection.

This study aimed to develop a systematic sensory lexicon for both gluten-containing and gluten-free chocolate chip cookies using a diverse selection of products representative of the U.S. market. Cookies are among the most widely consumed gluten-free snack items [38,50], valued for their familiarity and widespread availability [51]. Their long shelf life, affordability, and convenience make them a favored snack among individuals with celiac disease [52]. In addition to creating the lexicon, this study also sought to determine whether trained panelists could distinguish sensory differences between gluten-free and gluten-containing cookies. Given the known sensory disparities, especially in flavor and texture, between gluten-free and conventional baked goods [22], a deeper understanding of these differences may guide formulation improvements. Descriptive sensory analysis provides an objective framework for characterizing such differences and aligning product reformation efforts with consumer expectations [49,53].

## 2. Materials and Methods

The protocol of this study (1305713/2311502798) was approved by the University of Arkansas’ Institutional Review Board (IRB) in Fayetteville, AR, USA. Prior to participation, the experimental procedure was explained to all participants, and a written consent indicating voluntary participation was obtained from each participant.

### 2.1. Chocolate Chip Cookie Samples

A total of 24 chocolate chip cookie products (12 gluten-containing and 12 gluten-free) from 18 brands available in the U.S. market were evaluated in this study. The selection included both domestic and international brands and was informed by the products’ distinct sensory and ingredient profiles, insights from our prior research on the sensory attributes of chocolate chip cookies [54,55], and their accessibility to U.S. consumers. Additionally, five sensory professionals at the University of Arkansas Sensory Science Center (Fayetteville, AR, USA) sampled over 30 commercially available chocolate chip cookies and selected 24 products based on sensory diversity and ingredient composition.

All samples were purchased within two weeks of testing and stored at room temperature (approximately 21 °C) prior to descriptive sensory analysis. Table 1 summarizes the key characteristics of the selected samples. Based on product descriptions on packaging and brand websites, cookie samples were categorized as either “crispy” or “chewy.” “Shortbread” cookies were classified as “crispy,” while “soft” cookies were assigned to the “chewy” category. Textural properties of cookie samples were also evaluated using a texture analyzer (TA-XT2i, Stable Micro Systems, Godalming, UK) under test conditions adapted from Frauenhoffer et al. [55]. The primary textural parameter, maximum force (g), was measured via a compression test using a 6.25 mm diameter spherical probe. Pre-test, test, and post-test speeds were set at 1.0, 0.5, and 1.0 mm/s, respectively. Each sample was compressed to 70% strain to simulate the force required for human biting, which provides an objective measure of cookie hardness [56]. Four replicates per sample were tested. Maximum force values were analyzed using one-way analysis of variance (ANOVA) followed by post hoc comparisons with Tukey’s honest significant difference (HSD) test at a significance level of *p* < 0.05.

### 2.2. Descriptive Sensory Analysis of Cookie Samples

#### 2.2.1. Sensory Lexicon Development and Panel Training

Descriptive sensory analysis was conducted by seven professionally trained panelists at the University of Arkansas Sensory Science Center. Each panelist had over 1000 h of experience evaluating a broad range of food and beverage products, including chocolate chip cookies.

Lexicon development followed the Spectrum^TM^ method (Sensory Spectrum, Inc., Chatham, NJ, USA), as described by Dus et al. [57]. Panelists began by evaluating a broad range of chocolate chip cookies to generate an initial list of sensory attributes across five dimensions: aromas, flavors, basic tastes, textures, and residual sensory factors. Each panelist independently proposed descriptors, which were then compiled and refined through consensus discussions. Over two sessions totaling six hours, the panel collaboratively assessed various commercial cookie samples and finalized a set of 33 standardized sensory attributes (Table 2). Each term was clearly defined and paired with appropriate reference standards to ensure accuracy and consistency in subsequent evaluations [57].

Panelists subsequently underwent training to gain a thorough understanding of the reference standards and definitions for each sensory attribute. Conducted over four days (12 h in total), the training continued until panelists consistently and accurately applied both the attribute definitions and corresponding reference scales when evaluating the intensity of sensory characteristics in chocolate chip cookies.

#### 2.2.2. Procedure of Descriptive Sensory Analysis

Following the four-day training sessions (12 h), panelists evaluated the 24 chocolate chip cookie samples (12 gluten-containing (C) and 12 gluten-free (F) varieties) in a controlled sensory evaluation room under consistent white lighting. Evaluations were carried out over three days (three hours per day), with eight samples evaluated per day. After the initial round with all 24 samples, the evaluation process was repeated to allow duplicate assessments of each sample, resulting in a total of six days of testing.

Samples were presented in a randomized sequential monadic format. Depending on sample size, panelists received either 2–3 small cookies or 1–2 large cookies, served on white plastic plates. Evaluations were recorded using 10-inch tablets (Asus, Taipei, Taiwan) equipped with Compusense Cloud^®^ software (version 23.0.56, Compusense Inc., Guelph, ON, Canada). Panelists rated each sample across 33 predefined sensory attributes (Table 2) using a 0–15 scale with 0.1-point increments. To minimize sensory fatigue, at least 60 s were given between sample presentations. During these intervals, panelists were provided with spring water (Clear Mountain Spring Water, Taylor Distributing, Heber Springs, AR, USA) and unsalted crackers (Nabisco Premium Unsalted Tops Saltine Crackers, Mondelēz Global LLC., East Hanover, NJ, USA) for palate cleansing. A ten-minute break was also scheduled midway through each evaluation session.

### 2.3. Statistical Analysis

Data analysis was performed using JMP^®^ Pro (version 18, SAS Institute Inc., Cary, NC, USA) and XLSTAT (version 2023.3.0., Addinsoft, New York, NY, USA). To determine the global effect of “gluten” (C versus F), multivariate analysis of variance (MANOVA) was applied to compare intensity ratings for the 33 sensory attributes across the 12 cookie samples in each group (24 samples in total). To explore potential texture-dependent effects, separate MANOVAs were also conducted for cookies classified as “crispy” or “chewy.” (see Table 1). When MANOVA results indicated significance, mixed models were used to evaluate each sensory attribute individually, treating “gluten” as a fixed effect and “panel” and “session” as random effects. Post hoc comparisons were performed using Tukey’s HSD test. Statistical difference was defined at *p* < 0.05.

To reduce dimensionality and explore relationships among sensory attributes and cookie samples, principal component analysis (PCA) was conducted using the covariance matrix [49], appropriate for studies employing uniform measurement scales [55,58]. Agglomerative hierarchical clustering (AHC) was also conducted to identify patterns of associations among the cookie samples, and a dendrogram was generated to visually represent the clustering structure.

## 3. Results and Discussion

### 3.1. Sensory Lexicon of Gluten-Containing and Gluten-Free Chocolate Chip Cookies

Sensory lexicons are valuable communication tools that support various aspects of product development and quality control in the food industry [26,27,41,42,59]. In this study, we developed a standardized lexicon for both gluten-containing and gluten-free chocolate chip cookies, comprising 33 attribute terms: nine aromas, nine flavors, three basic tastes, seven textures, and five residual properties (Table 2). Each attribute was clearly defined and supported by reference materials and intensity standards.

While previous studies have proposed sensory lexicons for cookies, biscuits, or crackers [37,38,39], few have focused specifically on gluten-free cookies, especially those that represent commercially available products. For example, Jambrec et al. [33] developed a 13-attribute lexicon using a rapid method (i.e., free-choice profiling), covering appearance (color and color uniformity), the appearance/texture of the cross-section (crumbliness and sharpness), texture (hardness, fatness, fracturability, particle size or shape, and adhesiveness), odor (odor and off-odor), and taste (taste and off-taste). However, only two terms addressed flavor or odor, and basic taste attributes were absent. Their study also included only one gluten-free cookie produced in a pilot plant, with no indication of whether the other commercial products were gluten-containing or gluten-free [33], further reducing the utility of the lexicon [41].

In contrast, the lexicon developed in the present study was derived from a systematic evaluation of a broad sample of commercially available chocolate chip cookies, explicitly incorporating both C and F varieties. This comprehensive and rigorously developed lexicon offers a robust and versatile resource for sensory professionals and product developers in both academic and industry settings.

### 3.2. The Global Effect of Gluten Condition on Sensory Attributes of Chocolate Chip Cookies

We first examined whether C and F chocolate chip cookies differed significantly in their sensory attributes. A global MANOVA across all 33 attributes revealed a significant effect of gluten condition on intensity ratings among the 24 cookie samples (Wilks’ lambda = 0.73, *p* < 0.001), indicating that overall sensory profiles differed between the two groups (Table 3). Follow-up MANOVAs within each sensory module confirmed significant differences between C and F cookies in aroma (*p* < 0.001), flavor (*p* < 0.001), basic taste (*p* = 0.04), texture (*p* < 0.001), and residual property (*p* < 0.001) (Table 3). These findings suggest that all five sensory dimensions distinguish the twelve F cookies from their twelve C counterparts. Notably, these results are consistent with consumer-reported perceptions that gluten-free products differ noticeably in sensory characteristics from their gluten-containing equivalents [4,22,27,50,55].

**Figure 1:**
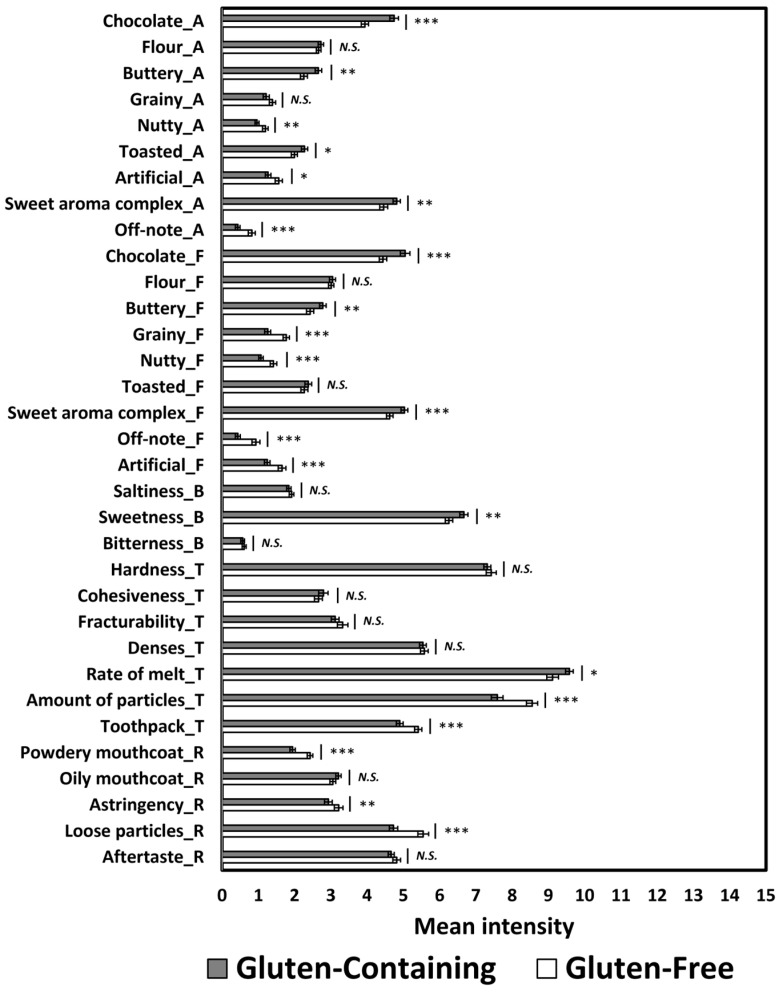
Mean intensity comparisons among gluten-containing (C) and gluten-free (F) cookies with respect to all 33 sensory attributes identified through descriptive analysis. *N.S*. represents no significant difference at *p* < 0.05. *, **, and *** represent a significant difference at *p* < 0.05, *p* < 0.01, and *p* < 0.001, respectively.

Following the MANOVAs, we conducted a mixed model analysis to further examine sensory attribute differences between C and F cookies. As shown in Figure 1, 21 attributes differed significantly between C and F cookies (*p* < 0.05), including seven aromas, seven flavors, one basic taste, three textures, and three residual properties. In terms of aroma, C cookies exhibited significantly higher intensities of chocolate, buttery, toasted, and sweet aroma complex, whereas F cookies were rated higher in nutty, artificial, and off-note aromas (*p* < 0.05). A similar pattern emerged in the flavor profiles. C cookies had stronger chocolate, buttery, and sweet aroma complex flavors, while F cookies were characterized by more pronounced grainy, nutty, artificial, and off-note flavors (*p* < 0.05). These differences were probably due to the use of alternative flours (e.g., buckwheat, cassava, and corn), which introduce flavor notes not typically associated with traditional chocolate chip cookies [60,61]. Gluten-free cookies often contain non-traditional ingredients to compensate for the lack of gluten, which may contribute to the higher levels of off-notes and artificial flavors observed [15,55]. Previous studies have shown that gluten-free cookies often receive lower flavor acceptability ratings due to such ingredient-driven variations [22,34,55].

For basic taste, C cookies were significantly sweeter than F cookies, while no significant differences were found in saltiness or bitterness (*p* > 0.05). The higher perceived sweetness in C cookies may be due to gluten’s ability to form a cohesive matrix, which can affect how sugar is distributed and released during chewing [62]. In contrast, gluten-free matrices, which differ in their water-binding and structural properties, may alter sweetness perception [50,62].

In terms of texture, C cookies melted more rapidly in the mouth, while F cookies received higher ratings for the amount of particles and toothpack attributes (*p* < 0.05). These differences likely stem from the absence of gluten’s binding capacity, which plays a key role in dough elasticity, cohesion [15,18,63,64], and moisture retention [3]. Although gluten-free formulations often incorporate hydrocolloids such as xanthan gum or guar gum as binding agents, they do not fully replicate gluten’s textural effects (e.g., elasticity and mouthfeel) [65,66]. Additionally, the crumbly and brittle texture commonly observed in F cookies is influenced by the type of flour used. For example, rice or maize flour tends to produce cookies with higher spread ratios, while sorghum or pearl millet flour yields lower spread ratios but higher fat, protein, and ash content, leading to increased firmness [5,67].

Finally, with respect to residual properties, F cookies exhibited significantly higher intensities of powdery mouthcoat, astringency, and loose particles compared to C cookies (*p* < 0.05), suggesting a drier and less cohesive texture [3]. While gluten’s structural role in cookies is less pronounced than in bread, due to the high fat and sugar content that limits gluten network formation, it still contributes to desirable texture, structure, and moisture retention, especially in firmer, semi-sweet, and sweet cookie varieties [15,50,68].

### 3.3. The Effect of Gluten Condition on Sensory Attributes of Chocolate Chip Cookies with Respect to Their Texture Categories: Crispy or Chewy

Chocolate chip cookies are a highly diverse product category, making them particularly suitable for sensory research and product reformulation [69]. Key ingredients such as flour, sugar, and fat play critical roles in determining the texture, flavor, and overall quality of cookies [70]. Given that chocolate chip cookies are commonly categorized as either crispy or chewy, additional global MANOVAs were conducted to determine whether sensory differences between C and F cookies persisted within each texture category. When analyzed separately by texture category, global MANOVAs revealed significant differences between C and F cookies in both the crispy and chewy groups (for all, *p* < 0.05; Table 3). In other words, C and F cookies differed significantly across all 33 sensory attributes, regardless of texture category. However, when the data were further analyzed by sensory module, no significant differences were observed between C and F cookies in the chewy group with respect to flavor (*p* = 0.21) or basic taste (*p* = 0.58).

As presented in Table 4, mixed model analysis identified 30 sensory attributes that significantly differed across the four subgroups: gluten-containing crispy, gluten-containing chewy, gluten-free crispy, and gluten-free chewy. However, no significant differences were observed for flour aroma (*p* = 0.68), oily mouthcoat (*p* = 0.07), and aftertaste (*p* = 0.11). Additionally, post hoc analysis revealed no significant differences among the subgroups in bitterness (*p* > 0.05).

In the crispy category, C cookies consistently exhibited significantly higher intensities of chocolate aroma/flavor and sweet aroma complex than F cookies. They also scored significantly higher in sweetness compared to F cookies (Table 4). In contrast, F cookies had significantly greater intensities of nutty aroma/flavor, off-note aroma/flavor, and grainy flavor than C cookies (*p* < 0.05). These sensory differences were likely due to the inclusion of non-traditional ingredients in gluten-free formulations, such as rice, oat, or nut flours, starches, gums, and flavor enhancers [5,15,55]. Many F cookies in this study contained ingredients commonly associated with nutty or grainy notes, such as flaxseed (FN, FP, FR), coconut (FN, FR), brown rice (FO), buckwheat (FQ), almonds (FR), and nuts (FR), which were more pronounced in crispy than chewy cookie samples.

However, such significant differences between C and F cookies in those attributes were not observed in the chewy group (*p* > 0.05). This may be due to differences in moisture content and baking conditions. Crispy cookies typically have lower water activity and longer baking times, which promote sugar caramelization and Maillard reactions that enhance sweet aroma complexity [71,72]. Chewy cookies, which retain more moisture, may suppress aroma and flavor release, thus reducing perceptible differences between C and F cookies.

Interestingly, toasted aroma was significantly higher in C cookies than in F cookies within the chewy group, but not the crispy group. Toasted notes are generally associated with Maillard browning reactions (e.g., formation of pyrazines) [73], which may be more efficiently developed in gluten-containing matrices due to their cohesive protein structure. In chewy cookies, longer internal bake times and higher moisture levels may still allow for such browning, while crispy cookies may dry too quickly for notable differences to emerge [74].

With respect to textural attributes, F cookies were rated significantly higher in hardness and fracturability than C cookies in the crispy group (Table 4), aligning with prior research that evaluated these attributes in crispy cookies [75,76]. Formulations with rice or nut/seed flour have been associated with increased hardness [3,76]. However, our instrumental texture analysis using a texture analyzer showed no significant difference in maximum force between C and F cookies in the crispy group (*p* = 0.24), although F cookies exhibited higher mean values than C cookies (C = 3483 g versus F = 4008 g). Notably, no significant differences between C and F cookies were observed in either sensory hardness (*p* > 0.05) or instrumental texture (*p* = 0.27) in the chewy group. However, cohesiveness, a key texture attribute in chewy cookies, was rated significantly higher in C cookies than in F cookies. This is likely due to gluten’s contribution to dough elasticity and structural integrity [15,18,63,75]. Additionally, across both texture categories, F cookies received significantly higher ratings for the amount of particles and loose particles (Table 4). This may be due to the lack of gluten and the use of coarse, non-traditional ingredients such as whole grains and nuts, which tend to create more particulate textures when chewed.

**Figure 2:**
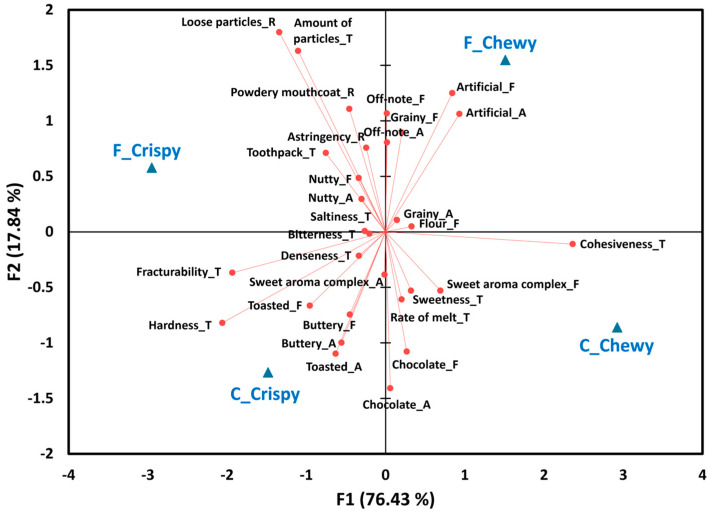
A biplot of principal component analysis, accounting for 94.27% of the total variance, was drawn based on significant sensory attributes of gluten-containing (C) and gluten-free (F) cookies as a function of texture category (crispy or chewy). The “A,” “F,” “T,” and “R” next to sensory attributes represent aroma, flavor, basic taste, texture, and residual property, respectively.

A biplot of the PCA, which accounted for 94.27% of the total variance, illustrates the associations between sensory attributes and both gluten condition (C versus F) and texture category (crispy versus chewy) (Figure 2). The first principal component (F1), explaining 76.43% of the variance, primarily distinguished cookies based on texture, separating crispy from chewy samples and associating with attributes such as hardness, fracturability, and cohesiveness. The second principal component (F2), accounting for 17.84% of the variance, reflected differences based on gluten content and was associated with attributes including aroma and flavors of chocolate, sweet aroma complex, and off-note. As shown in Table 4 and Figure 2, C cookies were more strongly associated with aromas and flavors of chocolate, a sweet aroma complex, toasted notes, sweetness, and rate of melt. In contrast, F cookies were more closely linked to off-note, artificial, grainy, and nutty aromas/flavors, as well as higher ratings for the amount of particles, toothpack, loose particles, powdery mouthcoat, and astringency. Notably, Figure 2 indicates that the texture category (crispy versus chewy) had a greater influence on the sensory attributes of chocolate chip cookies than the gluten condition.

### 3.4. Classification of Gluten-Containing and Gluten-Free Chocolate Chip Cookies Within Crispy and Chewy Categories

To evaluate whether F cookies can compete with C counterparts in terms of sensory attributes, we classified all 24 cookie samples (12 C and 12 F) based on similarities and differences in their sensory profiles. Given that texture type (crispy versus chewy) had a strong influence on sensory profiles (Figure 2), the classification was performed separately within each texture group.

Table 5 summarizes the results of mixed model analyses showing significant sensory differences among the 16 crispy and 8 chewy cookie samples. Supplementary Tables S1–S10 provide detailed results for each sensory module within each texture group.

**Figure 3:**
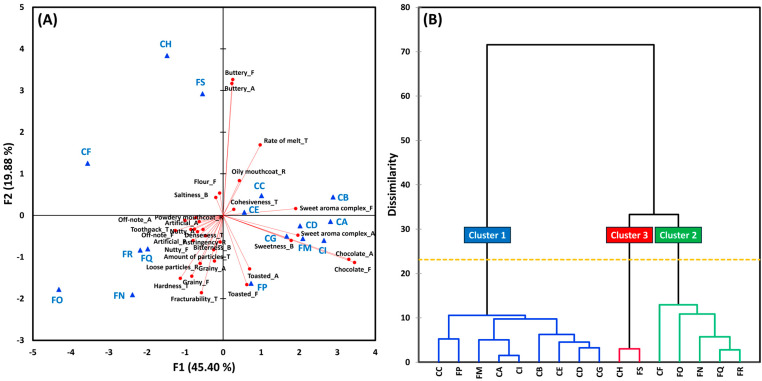
A biplot of principal component analysis (**A**) and a dendrogram of agglomerative hierarchical clustering analysis (**B**) based on 31 sensory attributes that differed significantly among the 16 crispy chocolate chip cookie samples. The “A,” “F,” “B,” “T,” and “R” next to sensory attributes represent aroma, flavor, basic taste, texture, and residual property, respectively.

Among the 16 crispy cookies (9 C and 7 F), all sensory attributes differed significantly (*p* < 0.05) except flour aroma (*p* = 0.47) and aftertaste (*p* = 0.55). Based on these significant attributes, both PCA and AHC were conducted to identify patterns among the samples. As shown in the PCA biplot and AHC dendrogram (Figure 3), the 16 crispy cookies were grouped into three distinct clusters. MANOVA identified significant differences among the clusters across the 33 sensory attributes [Wilks’ lambda = 0.12, *p* < 0.001].

Cluster 1 included most of the C cookies (CA, CB, CC, CD, CE, CG, and CI), along with two F cookies (FM and FP). These cookies were closely associated with C cookies’ typical attributes, such as aromas/flavors of chocolate and sweet aroma complex, sweetness, and rate of melt. This suggests that FM and FP were more similar to C cookies than to other F cookies in their sensory characteristics. Notably, while most F cookies in this study were vegan and plant-based (Table 1), FM and FP contained dairy ingredients, similar to the C cookies. Dairy proteins such as egg white and casein help reduce surface tension, stabilize foams at the gas–liquid interface, and retain air during baking, contributing to a more cohesive and aerated structure [2,77]. FM and FP cookies also showed comparable intensities of chocolate and sweet aroma complex to C cookies. These findings suggest that incorporating dairy ingredients and enhancing chocolate and sweetness-related flavors may be critical strategies for developing competitive F cookies within the crispy category.

Cluster 2 included most of the remaining F cookies (FN, FO, FQ, and FR) and one C cookie (CF). This group was characterized by sensory notes typical of gluten-free cookies, including grainy, nutty, and off-note aromas/flavors, along with higher ratings for hardness, fracturability, denseness, amount of particles, loose particles, and powdery mouthcoat. Sample CF, which contained no added sugar, exhibited reduced chocolate aroma/flavor and sweetness, along with elevated off-notes, explaining its classification within the gluten-free-dominant cluster.

Cluster 3 consisted of CH and FS. Both were shortbread-type cookies (Table 1), and their proximity in the PCA and AHC plots appears to be driven by their strong buttery aroma/flavor profiles, a known sensory characteristic of shortbread formulations [78].

**Figure 4:**
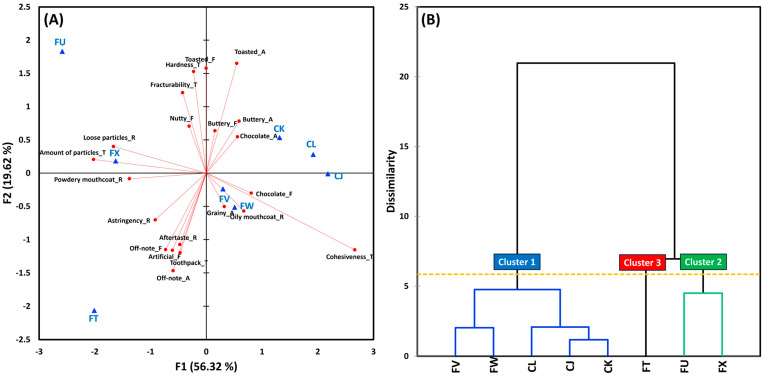
A biplot of principal component analysis (**A**) and a dendrogram of agglomerative hierarchical clustering analysis (**B**) based on 21 sensory attributes that differed significantly among the 8 chewy chocolate chip cookie samples. The “A,” “F,” “T,” and “R” next to sensory attributes represent aroma, flavor, basic taste, texture, and residual property, respectively.

For the eight chewy cookie samples (3 C and 5 F), 21 sensory attributes showed significant differences: five related to aroma, six to flavor, five to texture, and five to residual property (for all, *p* < 0.05). Based on these significant attributes, PCA and AHC analyses (Figure 4) grouped the chewy cookie samples into three clusters. MANOVA confirmed significant differences among the clusters across 33 sensory attributes [Wilks’ lambda = 0.17, *p* < 0.001].

Cluster 1 included all three C cookies (CJ, CK, and CL) and two F cookies (FV and FW). This group was associated with C cookies’ typical sensory characteristics such as chocolate and buttery aromas/flavors, cohesiveness, and oily mouthcoat. Similar to FM and FP in the crispy group, FV and FW may benefit from enhanced chocolate flavor to better compete with their C counterparts in the chewy category.

Cluster 2 comprised two F cookies (FU and FX), which were closely associated with higher levels of loose particles, the amount of particles, and powdery mouthcoat. These cookies were made with vegan, plant-based ingredients and gluten-free flours, which likely lacked sufficient binding capacity to form a cohesive matrix [2,77], resulting in a crumbly texture and more residual particles.

Lastly, Cluster 3 contained a single F sample (FT), which was defined by elevated off-note aroma/flavor and artificial flavor, as well as high intensities of toothpack, astringency, and aftertaste. These attributes are generally perceived as negative by consumers [54,55].

### 3.5. Implications and Future Studies

The findings of this study have meaningful implications for the food industry, particularly for manufacturers of C and F chocolate chip cookies. Understanding the sensory attributes that differentiate gluten-free from gluten-containing cookies can support formulation strategies aimed at improving product quality and market competitiveness.

This study demonstrated that enhancing chocolate and sweet aroma complex flavors, along with overall sweetness, is a key strategy for creating F cookies that more closely resemble their C counterparts in sensory characteristics. Prior research has shown that gluten-free breads tend to exhibit greater aroma compound volatilization during baking compared to wheat-based breads [79], which may partially explain the reduced aroma intensities observed in F cookies of this study. In chocolate chip cookies specifically, chocolate inclusions significantly influence flavor, sweetness, and fat content, thereby affecting overall sensory perception and consumer acceptance [55,80]. Based on these insights, manufacturers may consider increasing the amount of chocolate or other sweetness-enhancing ingredients or modifying recipes and baking parameters to minimize aroma loss during baking in gluten-free formulations.

Importantly, the use of vegan and plant-based ingredients did not result in F cookies that matched the sensory characteristics of their C counterparts [2,77]. Nevertheless, considering that approximately 4% of the U.S. population identifies as vegan [81], further research is warranted to enhance the textural and residual sensory properties of gluten-free cookies made with plant-based ingredients. Manufacturers may need to adjust fat and sugar content or incorporate novel binding agents to better replicate the structural and sensory qualities of gluten-containing products [2,7,17].

Future research should focus on consumer acceptance testing to identify which sensory attributes most strongly influence overall liking and purchasing behavior [29,54,55]. While the present study identified distinct sensory differences between C and F cookies, a deeper understanding of consumer-driven preferences is essential to guide formulation improvements that resonate with target markets. For example, Frauenhoffer et al. [55] found that participants without celiac disease liked commercially available C cookies than their F counterparts in terms of flavor, texture, and overall impression. These aspects were also likely to influence the lower purchase intent for gluten-free alternatives. Similarly, Ola and Seo [54] reported that F cookies received lower acceptance scores, particularly for texture and overall impression, than their C counterparts.

Moreover, although many consumers without dietary restrictions also choose to consume gluten-free products [10,11,12,13], further research should investigate differences in sensory perception and acceptance between consumers who follow gluten-free diets (e.g., individuals with celiac disease) and those who do not [22]. This line of research could provide valuable insights into segment-specific preferences and inform targeted product development.

## 4. Conclusions

This study developed a comprehensive sensory lexicon comprising 33 attributes to characterize gluten-containing and gluten-free chocolate chip cookies. The lexicon was created based on a wide range of commercially available products in the U.S. and serves as a valuable communication tool to support sensory profiling and product development, regardless of gluten content. Descriptive sensory analysis of 24 cookie samples (12 C and 12 F) revealed significant differences in 21 of the 33 attributes evaluated. C cookies exhibited stronger chocolate, buttery, toasted, and sweet aroma/flavor profiles. In contrast, F cookies were characterized by more pronounced nutty, artificial, and off-note sensory attributes. Additionally, C cookies were perceived as sweeter, melted more readily in the mouth, and showed fewer negative texture attributes compared to F cookies, which received higher ratings for toothpack, powdery mouthcoat, and astringency. PCA and AHC analyses further demonstrated that cookie samples could be grouped into three clusters within each texture category (crispy or chewy). Notably, some F cookies closely resembled C cookies in sensory profiles, particularly when chocolate flavor, sweetness, and cohesive texture were emphasized. These findings highlight the potential for certain gluten-free formulations to effectively replicate the sensory characteristics of their gluten-containing counterparts. Overall, this study offers detailed sensory insights that can guide food manufacturers, product developers, and sensory professionals in creating more competitive, appealing, and consumer-acceptable gluten-free chocolate chip cookie products.

## Figures and Tables

**Table 1:** Gluten-containing (C) and gluten-free (F) chocolate chip cookie samples used in this study and their sample codes and characteristics.

Code	Characteristics ^1^							Dimension ^2^	Textural Parameter
	Crispy/Crunchy	Short Bread	Soft	Chewy	Vegan/ Plant-Based	No Sugar/ Sugar Free	Dia- Meter (mm)	Thickness (mm)	Maximum Force (g) ^3^
CA	○	–	–	–	–	–	72.46	9.60	3025.91 ^cde^
CB	○	–	–	–	–	–	70.04	9.69	1488.41 ^efg^
CC	○	–	–	–	–	–	41.01	6.81	2557.59 ^cdef^
CD	○	–	–	–	–	–	58.54	10.06	3420.21 ^c^
CE	○	–	–	–	–	○	50.55	11.46	5078.89 ^b^
CF	○	–	–	–	–	○	70.71	10.36	2833.40 ^cde^
CG	○	–	–	–	–	–	75.48	12.70	3101.22 ^cd^
CH	–	○	–	–	–	–	54.82	10.84	7186.26 ^a^
CI	–	–	–	○	–	–	55.73	9.42	2655.84 ^cdef^
CJ	–	–	–	○	–	–	58.42	11.20	1485.22 ^efg^
CK	–	–	○	–	–	–	56.68	10.84	3179.95 ^cd^
CL	–	–	○	–	–	–	58.49	12.77	820.28 ^g^
FM	○	–	–	–	–	–	71.99	8.88	1778.36 ^defg^
FN	○	–	–	–	○	–	71.73	9.70	3493.13 ^c^
FO	○	–	–	–	○	–	35.08	12.33	5168.68 ^b^
FP	○	–	–	–	–	–	53.37	14.69	3064.97 ^cd^
FQ	○	–	–	–	○	–	48.10	12.77	3490.56 ^c^
FR	○	–	–	–	○	–	45.42	9.78	7279.67 ^a^
FS	–	○	–	–	–	–	53.20	10.20	3783.10 ^bc^
FT	–	–	–	○	○	–	66.69	10.58	632.59 ^g^
FU	–	–	○	–	○	–	35.83	15.98	2442.44 ^cdef^
FV	–	–	○	–	–	–	48.46	13.42	1465.61 ^efg^
FW	–	–	○	–	○	–	45.47	12.89	1736.50 ^defg^
FX	–	–	○	–	○	–	45.80	11.58	1180.78 ^fg^

^1^ These characteristics were identified from the packaging or corresponding websites. ○ = Yes; – = No; gray shading indicates the “crispy” cookie group, while white indicates the “chewy” group. ^2^ For each sample, mean values were calculated based on measurements from three cookies. ^3^ For each sample, mean values were calculated from four individual cookie measurements. Means with different superscript letters indicate significant differences, determined by post hoc comparisons using Tukey’s Honest Significant Difference test at *p* < 0.05, following a one-way analysis of variance.

**Table 2:** A lexicon of chocolate chip cookies was developed in this study.

Term	Definition	Reference (Intensity)
***Aroma***		
Chocolate	The aroma associated with chocolate	Universal aromatic scale ^1^
Flour	The aroma associated with raw, cooked, or toasted flour	Universal aromatic scale
Buttery	The aroma associated with butter	Universal aromatic scale
Grainy	A general term used to describe the aroma of raw or cooked grains, which cannot be tied to a specific grain type	Universal aromatic scale
Nutty	The aroma associated with nuts	Universal aromatic scale
Toasted	The aroma associated with being toasted	Universal aromatic scale
Artificial	The aroma associated with artificial notes	Universal aromatic scale
Sweet aroma complex	The aroma associated with products that also have a sweet taste, such as molasses, caramelized sugar, maple syrup, honey, and vanilla	Universal aromatic scale
Off-note	The aroma associated with off-notes such as cardboard, barnyard, and oxidized	Universal aromatic scale
***Flavor***		
Chocolate	The aromatics associated with chocolate	Universal aromatic scale
Flour	The aromatics associated with raw, cooked, or toasted flour	Universal aromatic scale
Buttery	The aromatics associated with butter	Universal aromatic scale
Grainy	A general term used to describe the aromatics of raw or cooked grains, which cannot be tied to a specific grain type	Universal aromatic scale
Nutty	The aromatics associated with nuts	Universal aromatic scale
Toasted	The aromatics associated with being toasted	Universal aromatic scale
Sweet aroma complex	The aromatics associated with products that also have a sweet taste, such as molasses, caramelized sugar, maple syrup, honey, and vanilla	Universal aromatic scale
Off-note	The aromatics associated with off-notes, such as cardboard, barnyard, and oxidized	Universal aromatic scale
Artificial	The aromatics associated with artificial flavors	Universal aromatic scale
***Basic Taste***		
Saltiness	The basic taste, perceived on the tongue, stimulated by salt, especially sodium chloride	Sodium chloride solution: 0.2% = 2.0 0.35% = 5.0 0.55% = 10.0
Sweetness	The basic taste, perceived on the tongue, stimulated by sugars and high potency sweeteners	Sucrose solution: 2% = 2.0 5% = 5.0 10% = 10.0 16% = 15.0
Bitterness	The basic taste, perceived on the tongue, stimulated by solutions such as caffeine	Caffeine solution: 0.05% = 2.0 0.08% = 5.0 0.15% = 10.0 0.20% = 15.0
***Texture***		
Hardness	The force required to compress the sample (technique: compress or bite through the sample once with molars or incisors)	Cream cheese ^2^ = 1.0 Frankfurter A = 7.0
Cohesiveness	The amount the sample deforms rather than splits apart, cracks, or breaks (technique: place the sample between the molar teeth and compress fully. May also be performed with incisors; scale: crumbles to deforms)	Corn muffin ^3^ = 1.0 Am. cheese = 5.0 Soft pretzel = 8.0
Fracturability	The force with which the sample breaks (technique: compress or bite through the sample once with molars or incisors; scale: crumbles to fractures)	Corn muffin ^4^ = 1.0 Graham crackers = 2.5 Melba toast = 6.7 Ginger snaps = 8.0 Pita chips = 10.0
Denseness	The compactness of a cross-section (technique: compress or bite through a sample once with molars or incisors; scale: airy to dense)	Nougat center ^5^ = 4.0 Malt balls = 6.0 Frankfurter B = 9.5
Rate of melt	The rate at which the product melts during chewing/manipulation (technique: chew the sample with molars until phase changes; scale: slow to fast)	25–30 chews = 2.0 20–24 chews = 5.0 15–19 chews = 7.5 10–14 chews = 10.0 5–9 chews = 12.0 0–4 chews = 15.0
Amount of particles	The relative number (or amount) of particles in the mouth after chewing the sample (technique: chew the sample up to ten times and evaluate)	Pudding + cornmeal ^6^ = 2.5 Pudding + cornmeal ^7^ = 6.0 Pudding + cornmeal ^8^ = 8.0 Pudding+ cornmeal ^9^ = 10.0
Toothpack	The amount of product packed into the crowns of the teeth after mastication (technique: chew the sample up to 15–20 times, expectorate, and feel the surface of the crowns of the teeth to evaluate)	Graham crackers ^10^ = 3.5 Cap’n Crunch = 5.0
***Residual Property***		
Loose particles	The amount of particles remaining in and on the surface of the mouth after swallowing (technique: chew the sample with molars, swallow, and evaluate)	Apple ^11^ = 3.0 Baby carrot = 10.0
Powdery mouthcoat	The amount of powdery residue felt by the tongue when moved over the surface of the mouth (technique: chew the sample up to 15 times and expectorate. Feel the surface of the mouth with the tongue to evaluate the amount of powder in the sample)	
Oily mouthcoat	The amount and degree of residue felt by the tongue when moved over the surface of the mouth (technique: expectorate the sample and feel the surface of the mouth with the tongue to evaluate)	Whole milk ^12^ = 3.0 Syrup = 6.0 Heavy cream = 7.5
Astringent	The residual feeling factor on the tongue or other skin surfaces of the mouth is described as puckering or drying (technique: swish the sample in the mouth, expectorate, and wait 30 s)	Alum solution 0.01% = 6.0
Aftertaste	Intensity of the flavor that remains in the mouth after swallowing (technique: measure the intensity of the remaining flavor in the mouth 15 s after swallowing)	Universal Aromatic Scale

^1^ Soda note in Nabisco Premium Unsalted Tops Saltine Crackers (Nabisco, East Hanover, NJ, USA) = 2.0; cooked-apple note in Mott’s Applesauce (Dr. Pepper Snapple Group, Plano, TX, USA) = 5.0; orange note in Minute Maid Frozen Concentrate Orange Juice (Coca-Cola, Atlanta, GA, USA) = 7.5, reconstituted with 1065 mL of filtered water; grape note in Welch’s Concord Grape Juice (Welch’s, Concord, MA, USA) = 10.0. ^2^ American cheese (Boars Head, Brooklyn, NY, USA): cut into 1.27 cm cubes (Boars Head, Brooklyn, NY, USA); Frankfurter A (Hebrew National beef frank, ConAgra Foods, Indianapolis, IN, USA): boiled for 5 min and cut into 1.27 cm slices; ^3^ Jiffy corn muffin (Chelsea Milling Company, Chelsea, MI, USA): cut into 1.27 cm cubes; American cheese (Boars Head, Brooklyn, NY, USA): cut into 1.27 cm cubes (Boars Head, Brooklyn, NY, USA); Soft pretzel (J & J Snack Foods Corporation, Mount Laurel, NJ, USA): cut into 1.27 cm cubes; ^4^ Jiffy corn muffin (Chelsea Milling Company, Chelsea, MI, USA): cut into 1.27 cm cubes; Graham Crackers (Nabisco, East Hanover, NJ, USA): 1.27 cm squares; Ginger snaps (Nabisco, East Hanover, NJ, USA); Melba toast (B&G Foods, Parsippany, NJ, USA); Pita chips (Stacy’s, Randolph, MA, USA); ^5^ Nougat center of three musketeers bar (Mars, McLean, VA, USA): cut into 1.27 cm pieces; Malt balls (Hershey Company, Hershey, PA, USA); Frankfurter B (Oscar Meyer beef frank, Oscar Meyer, Madison, WI, USA): boiled for 5 min and cut into 1.27 cm pieces; ^6^ 236.59 mL chocolate pudding (Conagra, Chicago, IL, USA) + 2.46 mL yellow corn meal (Associated Wholesale Grocers, Kansas City, KS, USA); ^7^ 236.59 mL chocolate pudding (Conagra, Chicago, IL, USA) + 4.93 mL yellow corn meal (Associated Wholesale Grocers, Kansas City, KS, USA); ^8^ 236.59 mL chocolate pudding (Conagra, Chicago, IL, USA) + 7.39 mL yellow corn meal (Associated Wholesale Grocers, Kansas City, KS, USA); ^9^ 236.59 mL chocolate pudding (Conagra, Chicago, IL, USA) + 9.86 mL yellow corn meal (Associated Wholesale Grocers, Kansas City, KS, USA); ^10^ Graham Crackers, 2.54 cm squares (Nabisco, East Hanover, NJ, USA); Captain Crunch (Pepsico, Purchase, Harrison, NY, USA); ^11^ Pink lady apple, peeled and cut into 1.27 cm cubes; Baby carrots, cut into 1.27 cm cubes; ^12^ Whole milk (Harps Food Stores, Springdale, AR, USA); Log Cabin syrup (Pinnacle Foods, Parsippany-Troy Hills, NJ, USA); Heavy cream (Organic Valley, La Farge, WI, USA).

**Table 3:** Summary of multivariate analyses of variance (MANOVAs) comparing gluten-containing (C) and gluten-free (F) chocolate chip cookies across sensory attributes for all cookies (*n* = 24), crispy (*n* = 16), or chewy (*n* = 8) varieties.

Group	All Attributes	Aroma	Flavor	Basic Taste	Texture	Residual Property
All Cookies	0.73 ^1^ (<0.001)	0.88 (<0.001)	0.87 (<0.001)	0.98 (0.04)	0.92 (<0.001)	0.90 (<0.001)
Crispy cookies	0.60 (<0.001)	0.85 (<0.001)	0.84 (<0.001)	0.94 (0.005)	0.81 (<0.001)	0.91 (0.001)
Chewy cookies	0.47 (<0.001)	0.81 (0.007)	0.89 (0.21)	0.98 (0.58)	0.78 (<0.001)	0.76 (<0.001)

^1^ Wilks’ Lambda (*p*-value).

**Table 4:** Comparisons in sensory attribute intensities of chocolate chip cookie samples as a function of gluten condition and texture category.

Module	Attributes	Gluten-Containing (C) Cookies		Gluten-Free (F) Cookies		*F*-Ratio (*p*-Value)
		Crispy (*n* = 9)	Chewy (*n* = 3)	Crispy (*n* = 7)	Chewy (*n* = 5)	
Aroma	Chocolate	4.85 ^a^	4.43 ^ab^	3.90 ^b^	3.99 ^b^	10.90 (<0.001)
	Flour	2.72	2.74	2.61	2.73	0.50 (0.68)
	Buttery	2.83 ^a^	2.15 ^bc^	2.54 ^ab^	1.86 ^c^	12.46 (<0.001)
	Grainy	1.10 ^b^	1.57 ^a^	1.45 ^ab^	1.31 ^ab^	3.42 (0.02)
	Nutty	1.00 ^b^	0.86 ^b^	1.33 ^a^	1.00 ^b^	6.80 (<0.001)
	Toasted	2.37 ^a^	1.98 ^a^	2.45 ^a^	1.36 ^b^	19.81 (<0.001)
	Artificial	1.06 ^b^	1.91 ^a^	1.06 ^b^	2.27 ^a^	33.27 (<0.001)
	Sweet aroma complex	4.93 ^a^	4.46 ^ab^	4.33 ^b^	4.63 ^ab^	5.38 (0.001)
	Off-note	0.40 ^b^	0.54 ^ab^	0.76 ^a^	0.90 ^a^	5.65 (<0.001)
Flavor	Chocolate	5.05 ^a^	5.03 ^ab^	4.36 ^b^	4.55 ^ab^	4.92 (0.002)
	Flour	2.97 ^ab^	3.27 ^a^	2.87 ^b^	3.21 ^a^	3.94 (0.009)
	Buttery	2.95 ^a^	2.27 ^b^	2.60 ^ab^	2.19 ^b^	8.86 (<0.001)
	Grainy	1.11 ^b^	1.69 ^a^	1.73 ^a^	1.83 ^a^	10.36 (<0.001)
	Nutty	1.10 ^b^	0.99 ^b^	1.57 ^a^	1.20 ^b^	8.40 (<0.001)
	Toasted	2.59 ^a^	1.75 ^b^	2.74 ^a^	1.60 ^b^	23.19 (<0.001)
	Sweet aroma complex	5.03 ^a^	5.03 ^a^	4.44 ^b^	4.88 ^a^	7.19 (<0.001)
	Off-note	0.40 ^c^	0.54 ^bc^	0.85 ^ab^	1.05 ^a^	8.61 (<0.001)
	Artificial	1.04 ^b^	1.86 ^a^	1.18 ^b^	2.31 ^a^	28.56 (<0.001)
Basic taste	Saltiness	1.89 ^ab^	1.71 ^b^	2.05 ^a^	1.74 ^b^	4.40 (0.005)
	Sweetness	6.56 ^a^	7.01 ^a^	5.97 ^b^	6.66 ^a^	10.33 (<0.001)
	Bitterness	0.61 ^a^	0.45 ^a^	0.70 ^a^	0.47 ^a^	2.68 (0.047)
Texture	Hardness	7.72 ^b^	6.10 ^c^	8.45 ^a^	5.98 ^c^	87.14 (<0.001)
	Cohesiveness	2.09 ^c^	4.91 ^a^	1.97 ^c^	3.62 ^b^	101.31 (<0.001)
	Fracturability	3.53 ^b^	1.88 ^c^	4.20 ^a^	2.11 ^c^	47.40 (<0.001)
	Denseness	5.57 ^ab^	5.44 ^ab^	5.81 ^a^	5.26 ^b^	3.64 (0.01)
	Rate of melt	9.63 ^a^	9.42 ^ab^	8.95 ^b^	9.34 ^ab^	3.09 (0.03)
	Amount of particles	7.90 ^b^	6.66 ^c^	8.87 ^a^	8.10 ^b^	23.69 (<0.001)
	Toothpack	5.02 ^b^	4.54 ^b^	5.71 ^a^	4.99 ^b^	13.68 (<0.001)
Residual property	Powdery mouthcoat	2.08 ^b^	1.54 ^c^	2.43 ^a^	2.42 ^ab^	11.47 (<0.001)
	Oily mouthcoat	3.15	3.40	2.98	3.15	2.39 (0.07)
	Astringency	3.03 ^ab^	2.64 ^b^	3.16 ^a^	3.29 ^a^	4.52 (0.004)
	Loose particles	5.00 ^b^	3.90 ^c^	5.77 ^a^	5.24 ^b^	25.87 (<0.001)
	Aftertaste	4.69	4.58	4.70	4.98	2.02 (0.11)

Mean ratings with different superscripts within the same row indicate a significant difference at *p* < 0.05.

**Table 5:** *F*-ratios (*p*-value) of mixed model analyses that determined whether crispy or chewy chocolate chip cookie samples differed in 33 sensory attributes.

Module	Attributes	Crispy Cookies (*n* = 16)	Chewy Cookies (*n* = 8)
Aroma	Chocolate	17.70 (<0.001)	2.55 (0.02)
	Flour	0.99 (0.47)	0.82 (0.58)
	Buttery	12.33 (<0.001)	2.29 (0.03)
	Grainy	2.87 (<0.001)	2.14 (0.047)
	Nutty	2.90 (<0.001)	1.89 (0.08)
	Toasted	6.32 (<0.001)	4.65 (<0.001)
	Artificial	3.17 (<0.001)	1.68 (0.12)
	Sweet aroma complex	8.61 (<0.001)	1.73 (0.11)
	Off-note	4.24 (<0.001)	3.44 (0.003)
Flavor	Chocolate	17.27 (<0.001)	2.48 (0.02)
	Flour	2.21 (0.007)	1.47 (0.19)
	Buttery	15.57 (<0.001)	3.67 (0.002)
	Grainy	5.37 (<0.001)	1.10 (0.37)
	Nutty	4.82 (<0.001)	2.99 (0.007)
	Toasted	7.53 (<0.001)	3.27 (0.004)
	Sweet aroma complex	9.74 (<0.001)	1.20 (0.31)
	Off-note	5.18 (<0.001)	3.15 (0.005)
	Artificial	3.84 (<0.001)	2.23 (0.04)
Basic taste	Saltiness	2.60 (0.001)	1.22 (0.30)
	Sweetness	8.23 (<0.001)	0.89 (0.52)
	Bitterness	7.41 (<0.001)	0.95 (0.47)
Texture	Hardness	9.04 (<0.001)	2.87 (0.009)
	Cohesiveness	2.33 (0.004)	13.74 (<0.001)
	Fracturability	4.64 (<0.001)	3.54 (0.002)
	Denseness	3.59 (<0.001)	1.27 (0.27)
	Rate of melt	3.70 (<0.001)	0.52 (0.82)
	Amount of particles	2.90 (<0.001)	3.97 (<0.001)
	Toothpack	3.38 (<0.001)	3.81 (0.001)
Residual property	Powdery mouthcoat	4.12 (<0.001)	5.77 (<0.001)
	Oily mouthcoat	2.89 (<0.001)	3.49 (0.002)
	Astringency	2.60 (0.001)	2.23 (0.04)
	Loose particles	3.41 (<0.001)	5.97 (<0.001)
	Aftertaste	0.92 (0.55)	2.83 (0.01)

## Data Availability

The raw data supporting the conclusions of this article will be made available by the authors on reasonable request, in accordance with applicable laws and institutional policies.

## Supplementary Materials

The following supporting information can be downloaded at: https://www.mdpi.com/article/10.3390/foods14132233/s1, Table S1. Mean comparisons among the 16 crispy cookie samples with respect to aroma attributes; Table S2. Mean comparisons among the 16 crispy cookie samples with respect to flavor attributes; Table S3. Mean comparisons among the 16 crispy cookie samples with respect to basic taste attributes; Table S4. Mean comparisons among the 16 crispy cookie samples with respect to texture attributes; Table S5. Mean comparisons among the 16 crispy cookie samples with respect to residual property attributes; Table S6. Mean comparisons among 8 chewy cookie samples with respect to aroma attributes; Table S7. Mean comparisons among 8 chewy cookie samples with respect to flavor attributes; Table S8. Mean comparisons among 8 chewy cookie samples with respect to basic taste attributes; Table S9. Mean comparisons among 8 chewy cookie samples with respect to texture attributes; Table S10. Mean comparisons among 8 chewy cookie samples with respect to residual property attributes.
