# Supplementary material for: Descriptive Sensory Analysis of Gluten-Containing and Gluten-Free Chocolate Chip Cookies Available in the Marketplace
Source: Foods. 2025 Jun 25;14(13):2233. doi: 10.3390/foods14132233 (PMC12249148; doi:10.3390/foods14132233)
Supplement: Supplementary file 1 [file foods-14-02233-s001.zip › foods-3659175-supplementary.pdf]

Supplementary Table S1. Mean comparisons among the 16 crispy cookie samples with respect to aroma attributes

Sample	Chocolate	Flour	Buttery	Grainy	Nutty	Toasted	Artificial	Sweet aroma complex	Off-note
CA	5.64a	2.49	3.01bc	1.14abc	0.64b	3.48a	0.50b	5.86a	0.00b
CB	6.11a	2.51	2.79bc	0.79abc	0.93ab	2.57abcd	0.89b	4.86abcd	0.36b
CC	4.71ab	2.89	3.19b	1.04abc	1.21ab	2.07cde	1.07ab	5.00abcd	0.14b
CD	5.58a	2.79	2.11bc	1.22abc	1.01ab	2.19bcde	1.04ab	5.46ab	0.21b
CE	4.75ab	2.75	2.69bc	1.19abc	0.86ab	3.07abc	1.14ab	5.29abc	0.29b
CF	2.43c	2.43	2.43bc	1.71abc	1.11ab	1.71de	1.96a	3.86de	1.00ab
CG	5.35a	3.07	2.01c	1.00abc	0.96ab	1.79de	1.29ab	4.79abcd	0.57b
CH	3.18c	2.81	4.64a	0.64c	1.21ab	1.21e	0.86b	4.00cde	0.71b
CI	5.89a	2.76	2.57bc	1.18abc	1.04ab	3.25ab	0.75b	5.28abc	0.29b
FM	5.39a	2.21	2.86bc	0.68bc	1.21ab	2.75abcd	0.43b	5.71a	0.36b
FN	3.46bc	2.49	2.08bc	1.94ab	1.71a	2.57abcd	1.29ab	3.82de	0.65b
FO	2.93c	2.64	1.86c	1.37abc	1.15ab	2.14bcde	1.21ab	3.15e	1.82a
FP	5.15a	2.83	2.04bc	2.00a	0.89ab	2.25bcde	1.33ab	5.11abcd	0.43b
FQ	3.54bc	2.75	2.04bc	1.86abc	1.69a	2.61abcd	1.11ab	4.29bcde	0.79b
FR	3.17c	2.71	2.43bc	1.50abc	1.75a	2.79abcd	1.14ab	4.33bcde	0.57b
FS	3.68bc	2.65	4.46b	0.79abc	0.93ab	2.04cde	0.93b	3.93de	0.71b
F-ratio (*p*-value)	17.70 (< 0.001)	0.99 (0.47)	12.33 (< 0.001)	2.87 (< 0.001)	2.90 (< 0.001)	6.32 (< 0.001)	3.17 (< 0.001)	8.61 (< 0.001)	4.24 (< 0.001)

Supplementary Table S2. Mean comparisons among the 16 crispy cookie samples with respect to flavor attributes

Sample	Chocolate	Flour	Buttery	Grainy	Nutty	Toasted	Sweet aroma complex	Off-note	Artificial
CA	5.71abc	2.74ab	3.14bc	1.35bcd	0.79d	3.46ab	5.89a	0.14cd	0.43c
CB	6.79a	2.89ab	2.90bcd	0.64cd	1.29abcd	3.00abcd	5.14abc	0.50bcd	0.86abc
CC	5.00bcd	2.93ab	3.43b	1.00bcd	1.71abcd	2.56abcd	5.65ab	0.14cd	1.07abc
CD	5.61abc	3.15ab	2.11cde	1.36bcd	1.00cd	2.50bcd	5.29abc	0.29bcd	0.71bc
CE	4.79bcde	2.86ab	2.79bcde	1.46bcd	0.86cd	2.54bcd	4.82abcde	0.43bcd	1.68ab
CF	2.07g	2.93ab	2.50bcde	1.58bcd	1.22abcd	2.14cde	3.68ef	1.21ab	1.79a
CG	6.25ab	2.86ab	2.29cde	0.86bcd	0.93cd	2.07cde	4.86abcd	0.36bcd	1.21abc
CH	3.29efg	3.29a	4.75a	0.56d	1.29abcd	1.32e	4.57bcdef	0.50bcd	0.93abc
CI	5.97abc	3.11ab	2.64bcde	1.21bcd	0.82d	3.71a	5.39abc	0.00d	0.68bc
FM	5.68abc	2.21b	2.93bcd	1.00bcd	1.14bcd	3.39ab	5.36abc	0.21bcd	0.46c
FN	4.57cde	2.50ab	2.29cde	2.07ab	2.21a	3.07abc	3.79def	1.14abc	1.29abc
FO	2.79fg	2.79ab	2.04de	1.64bcd	1.57abcd	2.50bcd	3.61f	1.79a	1.43abc
FP	5.36abc	3.11ab	1.93de	1.75abcd	1.14bcd	2.19cde	5.46abc	0.21bcd	1.32abc
FQ	3.71def	2.89ab	2.61bcde	2.89a	1.86abc	3.04abcd	4.43cdef	0.93abcd	1.36abc
FR	3.79def	3.35a	1.75e	1.82abc	2.11ab	3.14abc	3.82def	1.00abcd	1.61ab
FS	4.61cde	3.22a	4.64a	0.93bcd	0.96cd	1.89de	4.64bcdef	0.64bcd	0.82abc
F-ratio (*p*-value)	17.27 (< 0.001)	2.21 (0.007)	15.57 (< 0.001)	5.37 (< 0.001)	4.82 (< 0.001)	7.53 (< 0.001)	9.74 (< 0.001)	5.18 (< 0.001)	3.84 (< 0.001)

Supplementary Table S3. Mean comparisons among the 16 crispy cookie samples with respect to basic taste attributes

Sample	Saltiness	Sweetness	Bitterness
CA	1.86ab	6.79abcde	0.43cd
CB	2.29ab	6.61abcde	1.50a
CC	1.89ab	7.14ab	0.50cd
CD	1.56ab	6.99abcd	0.64cd
CE	2.07ab	6.11bcdef	0.32cd
CF	1.93ab	5.21f	0.86abc
CG	1.71ab	7.57a	0.79bc
CH	2.18ab	5.83cdef	0.07d
CI	1.50b	6.75abcde	0.35cd
FM	1.90ab	7.00abcd	0.71cd
FN	2.18ab	5.29f	1.46ab
FO	1.64ab	5.75def	0.71cd
FP	1.93ab	7.07abc	0.43cd
FQ	2.29ab	5.61ef	0.44cd
FR	2.07ab	5.50ef	0.79bc
FS	2.32a	5.61ef	0.39cd
F-ratio (*p*-value)	2.60 (0.001)	8.23 (< 0.001)	7.41 (< 0.001)

Supplementary Table S4. Mean comparisons among the 16 crispy cookie samples with respect to texture attributes

Sample	Hardness	Cohesiveness	Fracturability	Denseness	Rate of melt	Amount of particles	Toothpack
CA	7.79bc	2.11ab	3.93abcd	5.25b	9.40abc	7.96abc	4.90abc
CB	7.22c	1.71b	2.96d	5.51b	10.18a	7.54bc	4.50c
CC	8.50b	1.75b	5.28a	5.79b	10.32a	7.96abc	4.96abc
CD	7.47bc	2.29ab	3.04d	5.15b	9.87a	7.58bc	5.39abc
CE	7.86bc	2.18ab	3.39bcd	5.65b	9.14abc	8.33abc	5.18abc
CF	7.29c	1.75b	3.11d	5.75b	10.04a	7.43c	4.96abc
CG	8.00bc	2.25ab	3.00d	6.07ab	8.64abc	8.17abc	4.79bc
CH	7.79bc	2.18ab	3.29cd	5.64b	9.46abc	7.89abc	5.71abc
CI	7.57bc	2.58a	3.75abcd	5.32b	9.66ab	8.22abc	4.79bc
FM	7.64bc	1.96ab	3.86abcd	5.68b	9.75ab	9.54a	5.75abc
FN	8.54b	1.75b	4.73abcd	5.96ab	7.61c	9.29ab	5.93ab
FO	10.21a	1.78b	5.18ab	6.79a	7.93bc	8.42abc	6.21a
FP	8.46b	2.00ab	5.04abc	5.43b	9.07abc	8.71abc	5.86ab
FQ	8.25bc	2.11ab	3.33bcd	5.46b	8.96abc	8.74abc	5.68abc
FR	8.39bc	2.04ab	4.18abcd	5.75b	9.64ab	8.39abc	4.93abc
FS	7.68bc	2.15ab	3.07d	5.57b	9.71ab	9.03abc	5.61abc
F-ratio (*p*-value)	9.04 (< 0.001)	2.33 (0.004)	4.64 (< 0.001)	3.59 (< 0.001)	3.70 (< 0.001)	2.90 (< 0.001)	3.38 (< 0.001)

Supplementary Table S5. Mean comparisons among the 16 crispy cookie samples with respect to residual property attributes

Sample	Powdery mouthcoat	Oily mouthcoat	Astringency	Loose particles	Aftertaste
CA	1.33d	3.32abc	2.68ab	5.25bc	4.65
CB	1.93cd	3.21abc	3.68a	4.50c	4.93
CC	2.07abcd	3.04abc	3.07ab	4.71bc	4.71
CD	2.39abc	3.04abc	2.96ab	5.18bc	4.36
CE	2.00bcd	3.21abc	2.93ab	5.04bc	4.89
CF	3.06a	2.54bc	3.57ab	5.07bc	4.86
CG	2.00bcd	3.42abc	3.32ab	5.07bc	4.64
CH	1.96bcd	3.54ab	2.57ab	4.67bc	4.68
CI	2.00bcd	3.00abc	2.47b	5.54abc	4.50
FM	2.07abcd	3.07abc	2.93ab	6.79a	4.71
FN	2.00bcd	3.28abc	3.57ab	5.57abc	5.21
FO	2.29abcd	2.50c	3.50ab	5.96ab	4.25
FP	2.64abc	2.79abc	2.85ab	5.47abc	4.51
FQ	2.54abc	2.79abc	3.11ab	5.68abc	4.86
FR	3.00ab	2.71bc	3.29ab	5.43abc	4.86
FS	2.46abc	3.75a	2.89ab	5.46abc	4.50
F-ratio (*p*-value)	4.12 (< 0.001)	2.89 (< 0.001)	2.60 (0.001)	3.41 (< 0.001)	0.92 (0.55)

Supplementary Table S6. Mean comparisons among 8 chewy cookie samples with respect to aroma attributes

Sample	Chocolate[1]	Flour	Buttery	Grainy[1]	Nutty	Toasted	Artificial	Sweet aroma complex	Off-note
CJ	4.44a	2.81	2.00ab	1.75a	1.00	1.93a	1.96	4.39	0.68b
CK	4.11a	2.93	2.21ab	1.79a	0.93	2.21a	1.85	4.46	0.50b
CL	4.76a	2.46	2.25a	1.19a	0.64	1.79a	1.92	4.54	0.43b
FT	3.79a	2.92	1.21b	1.68a	0.79	0.64b	2.86	4.25	1.79a
FU	4.29a	2.54	1.86ab	1.21a	1.29	1.83a	2.21	5.00	0.50b
FV	3.71a	2.96	2.36a	1.21a	0.82	1.36ab	1.97	4.64	0.50b
FW	4.50a	2.67	2.04ab	1.57a	1.18	1.29ab	1.82	5.00	0.68b
FX	3.66a	2.57	1.86ab	0.86a	0.93	1.68a	2.47	4.25	1.04ab
F-ratio (p-value)	2.55 (0.02)	0.82 (0.58)	2.29 (0.03)	2.14 (0.047)	1.89 (0.08)	4.65 (< 0.001)	1.68 (0.12)	1.73 (0.11)	3.44 (0.003)

[1] *Post hoc* comparisons using Tukey's Honest Significance Difference (HSD) test revealed no statistically significant differences among the cookie samples at $p < 0.05$.

Supplementary Table S7. Mean comparisons among 8 chewy cookie samples with respect to flavor attributes

Sample	Chocolate	Flour	Buttery	Grainy	Nutty	Toasted	Sweet aroma complex	Off-note	Artificial[1]
CJ	5.04ab	3.65	2.00ab	1.93	1.21ab	1.81ab	4.79	0.57ab	1.71a
CK	4.93ab	3.08	2.39ab	1.86	0.89b	2.01a	5.19	0.50b	1.68a
CL	5.14a	3.07	2.43ab	1.29	0.86b	1.43ab	5.11	0.54ab	2.19a
FT	4.64ab	3.51	1.71b	1.75	0.86b	0.86b	4.82	1.64a	2.89a
FU	4.36ab	3.11	2.11ab	2.04	1.65a	2.14a	4.82	0.61ab	1.85a
FV	4.89ab	3.43	2.79a	1.57	0.93ab	1.61ab	4.86	0.93ab	2.25a
FW	4.89ab	3.17	1.76b	2.07	1.32ab	1.40ab	5.21	0.57ab	1.89a
FX	3.96b	2.82	2.57ab	1.71	1.25ab	2.00a	4.68	1.50ab	2.68a
F-ratio (*p*-value)	2.48 (0.02)	1.47 (0.19)	3.67 (0.002)	1.10 (0.37)	2.99 (0.007)	3.27 (0.004)	1.20 (0.31)	3.15 (0.005)	2.23 (0.04)

[1]*Post hoc* comparisons using Tukey's Honest Significance Difference (HSD) test revealed no statistically significant differences among the cookie samples at $p < 0.05$.

Supplementary Table S8. Mean comparisons among 8 chewy cookie samples with respect to basic taste attributes

Sample	Saltiness	Sweetness	Bitterness
CJ	1.46	7.11	0.50
CK	1.75	6.89	0.29
CL	1.93	7.04	0.57
FT	1.75	6.61	0.50
FU	1.86	6.43	0.57
FV	1.50	7.04	0.21
FW	1.68	6.61	0.65
FX	1.89	6.61	0.43
F-ratio (p-value)	1.22 (0.30)	0.89 (0.52)	0.95 (0.47)

Supplementary Table S9. Mean comparisons among 8 chewy cookie samples with respect to texture attributes

Sample	Hardness	Cohesiveness	Fracturability	Denseness	Rate of melt	Amount of particles	Toothpack
CJ	6.35ab	5.47a	1.96ab	5.68	9.29	6.54bc	4.96abc
CK	6.25ab	4.76ab	1.79b	5.28	9.19	7.11abc	4.65abc
CL	5.71ab	4.50ab	1.89b	5.36	9.79	6.32c	4.00c
FT	5.42ab	3.07cd	1.61b	4.93	9.47	8.14abc	5.32ab
FU	6.57ab	1.86d	2.79a	4.71	9.50	8.64a	4.29bc
FV	5.96ab	5.11a	2.07ab	5.07	9.64	7.89abc	4.96abc
FW	5.29b	4.54ab	1.86b	6.01	9.43	7.39abc	4.75abc
FX	6.64a	3.54bc	2.21ab	5.57	8.68	8.43ab	5.64a
F-ratio (*p*-value)	2.87 (0.009)	13.74 (< 0.001)	3.54 (0.002)	1.27 (0.27)	0.52 (0.82)	3.97 (< 0.001)	3.81 (0.001)

Supplementary Table S10. Mean comparisons among 8 chewy cookie samples with respect to residual property attributes

Sample	Powdery mouthcoat	Oily mouthcoat	Astringency	Loose particles	Aftertaste
CJ	1.50c	3.25ab	2.64ab	3.54c	4.79ab
CK	1.71c	3.43a	2.79ab	4.32abc	4.54b
CL	1.39c	3.54a	2.50b	3.86bc	4.40b
FT	2.86ab	3.11ab	3.79a	4.96ab	5.57a
FU	3.04a	2.57b	3.31ab	5.68a	4.82ab
FV	2.25abc	3.61a	3.11ab	5.18ab	4.61ab
FW	1.86bc	3.50a	3.01ab	4.94ab	5.16ab
FX	2.11abc	2.96ab	3.25ab	5.46a	4.71ab
F-ratio (*p*-value)	5.77 (< 0.001)	3.49 (0.002)	2.23 (0.04)	5.97 (< 0.001)	2.83 (0.01)
